# Cryptographic key generation using deep learning with biometric face and finger vein data

**DOI:** 10.3389/frai.2025.1545946

**Published:** 2025-04-29

**Authors:** Tsehayu Gizachew Yirga, Hailu Gizachew Yirga, Eshetie Gizachew Addisu

**Affiliations:** ^1^Department of Computer Science, College of Computing and Informatics, Mekdela Amba University, Tulu Awlia, Ethiopia; ^2^Department of Computer Science, College Informatics, University of Gondar, Gondar, Ethiopia; ^3^Department of Information Systems, College of Informatics, University of Gondar, Gondar, Ethiopia

**Keywords:** deep learning, SNN, multimodal biometrics, post-quantum security, cryptography key

## Abstract

This research proposes a novel approach to cryptographic key generation using biometric data from face and finger vein modalities enhanced by deep learning techniques. Using pretrained models FaceNet and VGG19 for feature extraction and employing a Siamese Neural Network (SNN), the study demonstrates the integration of multimodal biometrics with fuzzy extractors to create secure and reproducible cryptographic keys. Feature fusion techniques, combined with preprocessing and thresholding, ensure robust feature extraction and conversion to binary formats for key generation. The model demonstrates impressive accuracy with a vector converter, achieving a sigma similarity of 93% and a sigma difference of 64.0%. Evaluation metrics, including False Acceptance Rate (FAR) and False Rejection Rate (FRR), indicate significant improvements, achieving FRR < 3.4% and FAR < 1%, outperforming previous works. Additionally, the adoption of Goppa code-based cryptographic systems ensures post-quantum security. This study not only enhances biometric cryptography’s accuracy and resilience but also paves the way for future exploration of quantum-resistant and scalable systems.

## 1 Introduction

In the age of digital transformation, ensuring the security of personal data has become more critical than ever. Traditional authentication systems, relying on passwords or PINs, are increasingly vulnerable to cyber-attacks and data breaches (Raja, 2024). Biometric authentication, which uses unique physical or behavioral traits such as fingerprints, face recognition, and vein patterns, offers a more secure and user-friendly alternative. Among these, the fusion of facial and finger vein biometrics has gained significant attention due to their robustness and accuracy. The process of extracting cryptographic keys directly from these biometric features, through techniques such as fuzzy extractors, promises a novel approach to secure data encryption and authentication (Akintoye and Akinwamide, 2024; Babu et al., 2024; Shamili Shanmugapriya et al., 2024).

Fuzzy extractors are cryptographic tools designed to generate secure keys from noisy biometric data. The concept leverages error-correcting codes to recover biometric traits even in the presence of minor variations, making them suitable for real-world applications where data are not always perfectly consistent. Traditional fuzzy extractors were primarily focused on single-modal biometric data, such as fingerprints or iris patterns. However, with the advancement of deep learning and multimodal biometrics, there is growing potential to combine face and finger vein recognition to generate more reliable and secure cryptographic keys (Shevchenko and Anikin, 2023; Kuznetsov et al., 2018).

Deep learning, particularly convolutional neural networks (CNNs), has revolutionized how we process and understand biometric data. Pretrained models such as FaceNet for face recognition and specialized CNNs for finger vein recognition have demonstrated exceptional performance in extracting discriminative features from biometric images. These features can then be fused to create a unique cryptographic key (Kuznetsov et al., 2022; Yao et al., 2024). This approach not only enhances the accuracy of biometric authentication but also improves the robustness of the generated keys, making them less susceptible to errors caused by environmental factors or user behavior.

Traditional authentication techniques, such as PINs and passwords, are susceptible to a number of online attacks. Although biometric authentication provides a more secure option, current methods for generating biometric cryptographic keys have issues with scalability, security, and consistency. Because of biometric variances, previous studies that concentrated on unimodal biometric data, such as fingerprints, faces, or iris, had high False Acceptance Rates (FARs) and False Rejection Rates (FRRs) (Bele, 2024).

This research introduces a multimodal biometric cryptographic key generation framework using deep learning. By fusing face and finger vein features, the proposed method enhances security and reliability over single-modal approaches. Additionally, incorporating a McEliece cryptosystem with Goppa codes ensures resistance against quantum computing attacks. The experimental results confirm the superiority of this method, achieving FRR < 3.4% and FAR < 1%, outperforming prior works that report FRR of 8.3% and FAR of 7.4%.

## 2 Related work

Biometric authentication has gained widespread attention as a robust alternative to traditional password-based security systems. Among various biometric modalities, face and finger vein recognition stand out due to their high accuracy, non-intrusiveness, and resilience to spoofing attacks. Face recognition has become a prominent method due to its ease of use and widespread availability through cameras in smartphones and other devices. Recent advancements in deep learning, particularly with models such as FaceNet, have significantly improved face recognition accuracy by learning discriminative features directly from large datasets (Sydor et al., 2024). Studies have reported a face recognition accuracy of 97.35% on the Labeled Faces in the Wild (LFW) dataset, showcasing the reliability of deep learning-based approaches. However, despite these improvements, face recognition is still susceptible to adversarial attacks, occlusions, and variations due to aging or environmental conditions, necessitating additional layers of security.

Studies have reported varying performance metrics for different biometric authentication methods. For instance, the work “Securing the Digital World: A Comprehensive Guide to Multimedia Security” achieved a False Rejection Rate (FRR) of 2.5% and a False Acceptance Rate (FAR) of 1.8%, demonstrating a strong balance between security and usability. Implementation and Analysis of Digital Watermarking Techniques for Multimedia Authentication reported an authentication accuracy of 96.4%, emphasizing the robustness of watermarking techniques for secure biometric verification. Secure and Imperceptible Frequency-Based Watermarking for Medical Images achieved a Peak Signal-to-Noise Ratio (PSNR) of 52 dB and a Structural Similarity Index (SSIM) of 0.98, indicating high imperceptibility and robustness against attacks. Additionally, Robust Medical and Color Image Cryptosystem Using Array Index and Chaotic S-Box recorded an encryption efficiency of 99.2%, ensuring secure transmission of biometric data while maintaining high image quality (Kavitha et al., 2024). These results highlight the effectiveness of different biometric security approaches, but challenges such as reproducibility, robustness against adversarial attacks, and computational efficiency remain significant concerns. Our work addresses these issues by integrating multimodal biometric fusion with optimized cryptographic key generation techniques, ensuring a more secure and scalable authentication system.

A previous study (Sulavko et al., 2025) presents a secure method for handling user-specific information using a Neural Fuzzy Extractor (NFE). The NFE integrates pre-existing classifiers with fuzzy extractors through an artificial neural network-based expander, maintaining performance while enhancing security. The reported FAR and FRR of 4.5% suggest a trade-off between security and usability. The authors demonstrate the NFE’s effectiveness by retrofitting it to classic neural networks for basic biometric authentication scenarios. However, the reliance on neural network-based expansion may introduce computational overhead, and further work is needed to explore its application to multimodal biometrics. Future research could focus on optimizing NFEs for different biometric traits and improving efficiency in large-scale deployments.

Another significant contribution comes from the article “A Secure Biometric Key Generation Mechanism via Deep Learning and Its Application” (Wang et al., 2022). This method utilizes random binary codes to represent biometric data and establishes a relationship between biometric data and the codes for each user. To protect privacy and ensure revocability, a random permutation operation shuffles the binary code to update a new biometric key. A fuzzy commitment module generates helper data without revealing biometric information during enrollment. The method is evaluated using benchmark datasets and outperforms existing methods in terms of the genuine accept rate at a 1% False Acceptance Rate, while also meeting revocability and randomness criteria. However, the need for network retraining limits its applicability in zero-shot enrollment scenarios, where users enroll without retraining the model. Future work should explore ways to improve stability and security under zero-shot conditions to enhance the practicality of biometric key generation systems.

Furthermore, the article “Deep Learning-based Biometric Cryptographic Key Generation with Post-Quantum Security” (Kuznetsov et al., 2023) explores convolutional neural networks for extracting biometric features from human facial images for key generation. Code-based cryptographic extractors process these features, resulting in a low error rate of less than 10%. Post-quantum cryptography enhances the security of generated keys, making them resilient to future computational threats. However, the study primarily focuses on facial biometrics, which alone may not provide the highest level of security due to potential vulnerabilities such as face spoofing. Future research should investigate the integration of multimodal biometrics and further optimization of code-based extractors to improve security and performance. Another method by De Oliveira Nunes et al. (2024) introduces oblivious extractors, which allow authentication without transmitting helper data (HD) to the client. While this approach enhances privacy by reducing the risk of HD interception, achieving suitable FRR and FAR rates below 10% remains a challenge. The effectiveness of this method could be improved by optimizing feature extraction techniques and integrating stronger cryptographic principles to mitigate statistical and reusability attacks.

Deep learning-based fuzzy extractors show promise for generating cryptographic keys from biometrics. They overcome the limitations of traditional methods and offer improved security and privacy. However, further research is needed to address challenges with accuracy and high error rates because of using unimodal to generate biometric keys. Therefore, we proposed code-based cryptography key generation using deep learning from multimodal biometrics.

Our research extends these works by integrating biometric multimodal fusion, deep learning, and quantum-resistant cryptography, ensuring a more secure and scalable authentication system. By employing FaceNet and VGG19 for feature extraction, a Siamese Neural Network (SNN) for pattern learning, and a McEliece cryptosystem for quantum-resistant security, our approach surpasses existing methods in terms of robustness, accuracy, and resilience against modern cyber threats. Unlike traditional methods that rely on either unimodal biometrics or non-optimized feature fusion, our approach ensures better generalization and resistance to environmental variations, making it suitable for real-world biometric authentication applications.

## 3 Methodology

### 3.1 Dataset

Biometric technologies, particularly face and finger vein recognition, have become key in security and authentication, supporting applications from personal devices to large-scale government systems. Their reliability and efficiency make them vital for enhanced security and seamless authentication. This research utilized a dataset from Kaggle, containing 425 images across 85 classes each for face and finger vein data.

### 3.2 Dataset preprocessing

To prepare face and fingerprint data for machine learning models, preprocessing is essential. Preprocessing for both face and fingerprint data can be carried out as follows:

#### 3.2.1 Preprocessing face dataset

Dataset preprocessing: Preprocessing is essential to prepare face and fingerprint data for machine learning models. Pre-trained Haar cascade classifiers were used to locate faces within images accurately. Detected face images were resized to a uniform dimension of 160 × 160 pixels in Red, Green, Blue (RGB) format. Gaussian blurring was applied to reduce background noise in face images, enhancing clarity and feature distinction. Each class was ensured to have exactly five images using the ImageDataGenerator from Keras. Various transformations were performed, including rotation, shear, zoom, and horizontal flipping, to generate additional samples for underrepresented classes and maintain uniformity across classes.

#### 3.2.2 Preprocessing finger vein dataset

In preprocessing of the finger vein dataset, the focus was on enhancing finger vein images using ROI extraction and contrast improvement. Each image was converted to grayscale, Gaussian blur was applied to reduce noise, and the Canny edge detector was used to extract the region of interest (ROI). The contrast of the extracted ROI was then enhanced using CLAHE (Contrast Limited Adaptive Histogram Equalization). This method ensures efficient and systematic preprocessing of finger vein data for subsequent analysis.

### 3.3 Feature extractor models

FaceNet was chosen for face feature extraction due to its performance in biometric applications. It creates complex feature vectors, or embeddings, that capture unique facial traits such as landmarks and expressions, which are crucial for differentiating individuals. To produce embeddings that preserve facial similarity and enable accurate face identification and verification, FaceNet uses a deep metric learning technique. It is a great option for challenging facial recognition tasks due to its adaptability in managing various stances, lighting scenarios, and expressions.

VGG19 was selected for fingerprint feature extraction because of its deep architecture, which consists of 19 layers, effectively learns intricate patterns in images, and captures subtle features such as ridges and valleys in fingerprints. It is the ideal choice for accurate fingerprint identification and classification because of its straightforward architecture, performance on large datasets, and dependable generalization to fresh fingerprint photographs. To further improve feature representation, Principal Component Analysis (PCA) is applied to the high-dimensional features that VGG19 has recovered. PCA reduces dimensionality by transforming the data into a more manageable 128-dimensional space and identifying and retaining the most significant primary components.

### 3.4 Feature fusion

Feature fusion is a technique that combines features from different modalities, such as face and fingerprint data, to create a more comprehensive representation for recognition tasks. This integration can occur at various stages in machine learning, including early fusion, where features are combined at the input level, and late fusion, where they are extracted separately and combined later. Early fusion enhances model performance by capturing connections between modalities simultaneously, requiring only one training step, making it more efficient than late fusion, which involves training multiple models.

#### 3.4.1 SNN model development

**Figure 1:**
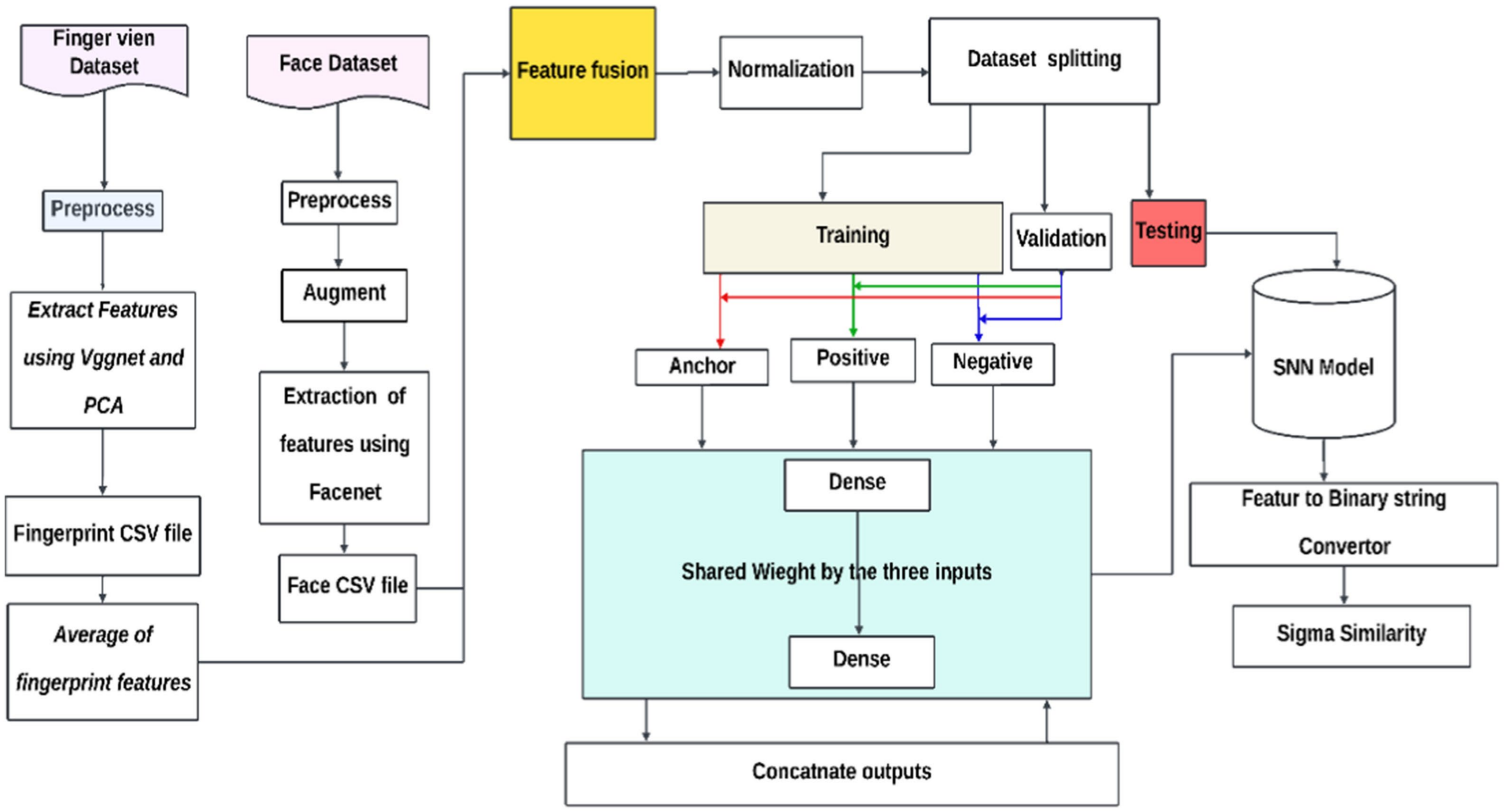
Development of the SNN model.

An architecture known as a Siamese Neural Network (SNN) is made for tasks that require learning or verifying similarity between three inputs: an anchor, a positive that is similar to the anchor, and a negative that is different from the anchor. Every input is routed via identical subnetworks with the same architecture and weights, producing high-dimensional embeddings for every input. The network maximizes the distance between different input embeddings and minimizes the distance between similar input embeddings using the triplet loss function. The architecture consists of two dense layers activated by sigmoid and Rectified Linear Unit (ReLU). The shared weights are tuned during training to discriminate between different and similar data points according to their embeddings. The end product is an SNN that can map comparable inputs more closely together while separating them in the embedding space, as shown in Figure 1.

### 3.5 Features of binary string converter

The function used for the feature vector converter is defined mathematically in Equation 1, which applies a thresholding rule to convert continuous real-valued features into binary format. One part of the extractor recommended for creating reliable keys from biometric photos is the feature vector converter. A feature vector with real-valued elements can be entered into the feature vector converter. Deep learning techniques extract the feature vector from the biometric images. The converter transforms the real-valued feature vector into a binary string using a binarization rule. A threshold value is used to define the binarization rule. The binarization rule compares each element of the feature vector with the threshold value. If an element is greater than the threshold, it is converted to 1; otherwise, it is converted to 0. The converter processes each element of the binarized feature vector and concatenates the binary values to form a binary string representation. The feature vector converter plays a crucial role in transforming the continuous real-valued features extracted from biometric images into a binary format. The binary distance between vectors is calculated using Equation 2, which computes the mean of the absolute differences between the elements of the two vectors. This binary representation is then used in the fuzzy extractor to generate cryptographically strong keys for authentication and security purposes. In general, we have used thresholds to convert the continuous real-valued features extracted from biometric images into a binary format. These are using zero thresholding and mean thresholding values. These values are compared with continuous real-valued features extracted from biometrics, and values below these are converted to zeros and values above these are converted to ones.

The similarity between binary vectors is determined using Equation 3, which is based on the binary distance.

The function used for the feature vector converter is:

The mathematical equation for the to_binary_string function can be written as


(1) $$B_{i}=\left\{\begin{array}{c} 1\;\mathrm{if}\;fi\geq ti\\ 0\;\mathrm{if}\;fi<ti \end{array}\right\}$$

Where $B_{i}$ is the *i*-th element of the binary output vector, fi is the *i*-th element of the input vector, and ti is the *i*-th element of the threshold vector t . This equation applies the rule *I* (*f > t*), meaning it returns 1 if the condition *f_i_* > =*t_i_* is satisfied and 0 otherwise for each element *i* in the vectors.

The equation for the binary distance as implemented in the code can be expressed mathematically as:


(2) $$D\left(x,y\right)=\frac{1}{n}\overset{n}{\underset{i=1}{\sum }}|x_{i}-y_{i}|$$

Where *D* (*x*, *y*) is the binary distance between vectors *x* and *y*, *n* is the number of elements in each vector, *x_i_* and *y_i_* are the corresponding elements of vectors *x* and *y*, and ∣*x_i_* − *y_i_*∣ is the absolute difference between the elements *x_i_* and *y_i_*. This equation computes the mean of the absolute differences between the elements of the two vectors.

Here is the equation for the similarity based on the binary distance:


(3) $$S\left(x,y\right)=1-D\left(x,y\right)$$

Where *S* (*x*, *y*) is the similarity between vectors *x* and *y* and *D* (*x*, *y*) is the binary distance between the vectors, which is computed above.

### 3.6 Evaluation parameters

The average similarity for items from the same class is calculated using Equation 4, which aggregates similarity scores across feature vectors. When evaluating the precision and efficacy of a system, model, or procedure, evaluation parameters are essential. They offer measurable metrics that aid in assessing a system’s performance in many scenarios. These criteria are crucial for determining one’s advantages, disadvantages, and potential growth areas. Researchers and developers can optimize the performance of their systems by examining these metrics. In this study, we measured the effectiveness of our biometric authentication system using a number of evaluation factors.

Similarity evaluation ( $\hat{\sigma }$ same): This parameter represents the average similarity between binary vectors formed for pairs of the same individual. It measures how closely the binary strings extracted from biometric data of the same person match each other. Higher values of $\hat{\sigma }$ same indicate a higher degree of similarity between the binary vectors, which is desirable for accurate authentication.

For a batch of feature vectors from the same class, the sigma_same (similarity between pairs of vectors in the same class) is calculated as:The average similarity for items from different classes is computed using Equation 5, which measures cross-class similarity.


(4) $$\hat{\sigma }\;\mathrm{same}=\frac{1}{\mathrm{Nsame}}\overset{C\;}{\underset{i=1}{\sum }}\overset{M}{\underset{j=1}{\sum }}sim\left(f_{j},f_{j+1}\right)$$

Hère: $\hat{\sigma }$ same is the averaged similarity (sigma) for items from the same class, *C* is the total number of unique classes, *M* is the number of items in the batch corresponding to class *C* (i.e., the number of vectors in that class), $sim\left(f_{j},f_{j+1}\right)$ is the similarity function applied to consecutive pairs of feature vectors, and Nsame is the total number of same-class pairs summed over all classes, which is equal to the sum of *M*−1 for each class.

Similarity evaluation ( $\hat{\sigma \;}$ diff): This parameter represents the average similarity between binary vectors formed for pairs of different individuals. It measures the level of similarity between the binary strings extracted from the biometric data of different individuals. Lower values of $\hat{\sigma \;}$ diff indicates a higher degree of dissimilarity between the binary vectors, which is important for distinguishing individuals.


(5) $$\hat{\sigma }\;\mathrm{diff}=\frac{1}{\mathrm{Ndiff}}\overset{C-1}{\underset{i=1}{\sum }}\overset{\min\left(Mi,Mi+1\right)-1}{\underset{j=1}{\sum }}sim\left(f_{i,j},f_{i+1,j}\right)$$

Hère: $\hat{\sigma }$ diff is the averaged similarity (sigma) for items from different classes. *C* is the total number of unique classes. *Mi* and *Mi* + 1 are the number of vectors in class *i* and class *i* + 1, respectively. $sim\left(f_{i,j},f_{i+1,j}\right)$ is the similarity between vectors from class i and class i + 1. Ndiff is the total number of cross-class pairs considered, which is equal to the sum of $\min\left(Mi,Mi+1\right)-1$ over all adjacent class pairs.

By using these two parameters, ( $\hat{\sigma }$ same) and ( $\hat{\sigma }$ diff) can comprehensively evaluate the performance of the biometric authentication system. High ( $\hat{\sigma \;}$ same) values ensure the system reliably recognizes the same individual, enhancing the true positive rate. Conversely, low ( $\hat{\sigma \;}$ diff) values ensure that the system effectively distinguishes between different individuals, reducing the false positive rate. Together, these parameters help balance the trade-off between security and convenience in biometric authentication systems.

### 3.7 Code-based fuzzy extractor for biometric cryptography

The Code-Based Fuzzy Extractor for Biometric Cryptography is a cryptographic method that uses the McEliece code-based cryptosystem. It can extract error-tolerant, nearly uniform randomness (K) from biometric data (w) and recover it from an analogous input (w’). K can be used as a cryptographic key without the requirement for conventional key storage thanks to this technique, which integrates information-theoretic security with cryptographic systems, even though computational security is frequently relied upon in such applications (Kuznetsov et al., 2018).

**Figure 2:**
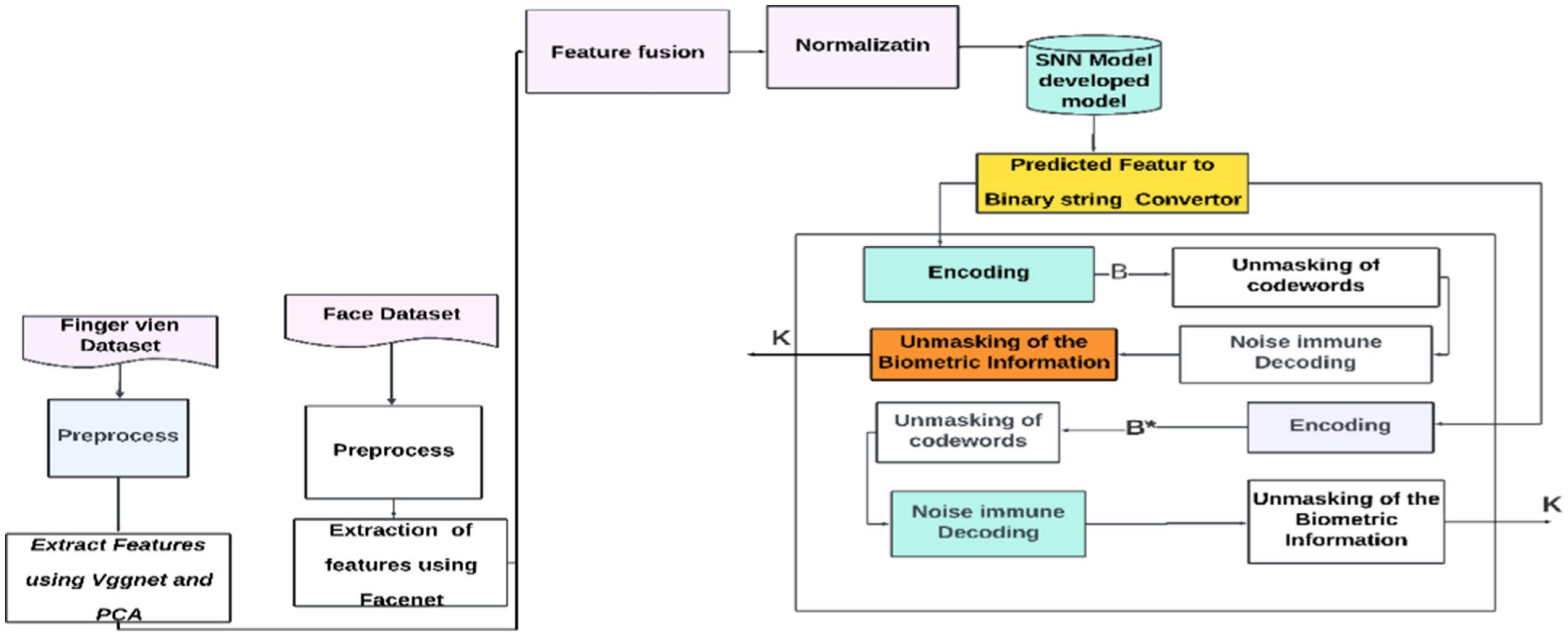
Developed SNN model with code-based cryptography.

By leveraging the strength and resilience of the McEliece cryptosystem against quantum cryptanalysis, the suggested fuzzy extractor enhances the security of biometric cryptography. The extractor ensures accuracy even in errors by correcting biometric image distortions. By doing away with the requirement for a non-secret helper string, it also streamlines the key creation procedure. Even with cutting-edge quantum computing technology, the extractor should be immune to quantum cryptanalysis, making it appropriate for safe cryptographic key production. To ensure a dependable and secure technique for key extraction from biometric data, the study investigates the balance between False Rejection Rate (FRR) and False Acceptance Rate (FAR) in biometric key creation. The McEliece cryptosystem, named after its creator, Robert McEliece, uses error-correcting codes, specifically Goppa codes, for public-key encryption. The security of the McEliece cryptosystem relies on decoding random linear codes, which are impervious to attacks such as factoring or discrete logarithm-based attacks. The proposed fuzzy extractor offers a secure and efficient approach to encrypting and decrypting messages, as shown in Figure 2.

The McEliece cryptosystem uses the private key, consisting of the inverses of matrices S and P (denoted as S_inv and P_inv), along with the Goppa code used to generate the public key using Equation 6. To encrypt a message, the sender multiplies the message by the public key Gx, creating a codeword using Equation 7. Random errors are then introduced at t locations to enhance security. The encrypted codeword is sent to the recipient, who uses S_inv and P_inv to decode it. The recipient first unshuffles the cipher using P_inv using Equation 8, decodes it with the Goppa code, and finally unscrambles it with S_inv to using Equation 9 retrieve the original message. The security of McEliece comes from the difficulty of decoding random linear codes, which resists attacks such as factoring or discrete logarithm-based methods. The system’s challenge lies in determining the generator matrix of a code from the code itself. As an efficient and secure approach to public-key encryption, the McEliece cryptosystem exemplifies robust key generation, encryption, and decryption processes.

Select a Goppa code using the given parameters (*n*, *k,* and *t*): *n* represents the length of the codeword, *k* represents the dimension of the code, and *t* represents the error-correcting capabilities of the code.

Public key k x n matrix *Gx* is generated by:


(6) $$G_{x}=G_{p}.S.P$$

Encoding the biometric data using the public key and introducing *t* errors, *I* is *k* bit information of the biometric.


(7) $$B_{x}^{*}=I.G_{x}+e$$

Where $B_{x}=I.G_{x}$ is the codeword of masked code with a generator matrix *G_P_*, *I* is the k-bit public text or biometric information, vector e is the secret error vector with hamming weight (number of non-zero positions) that equals wH(e) = t.

Decoding:


(8) $${\vec{B}}_{x}^{*}=B_{x}^{*}.P_{-inv}=I^{\prime }.G_{p}+e^{\prime }$$

In addition, decoded it to obtain $I^{\prime }$ .


(9) $$I=I'.S_{-inv}$$

then *I* is generated. Here, *I* is the password, which is expressed above as *K*.

#### 3.7.1 Initial registration

This is the process where information (biometric information) is converted into a particular form or codeword using a public key known as encoding. Unmasking of codeword: This step involves revealing or interpreting the encoded data using the secret keys. Noise immune decoding: This implies that the codeword can be decoded in the presence of some noise or interference using the Goppa code.

#### 3.7.2 Usage stage

Encoding: Once again, this represents the encoding of information using the public key of the data provided by users. Unmasking of information: Similar to the “Unmasking of codeword” in the first diagram, it might involve revealing the encoded information. Noise immune decoding: As before, it suggests that the information can be decoded notwithstanding noise interference.

Here, *B* and B* belong to the same person as defined in Figure 1 and in our interpretation, $B=I.G_{x}+e$ and $B^{*}=I.G_{x}+e^{*}$ (3). If ⅇ and e* are distinct vectors with a Hamming weight lower than *t*, then decoding the vectors will result in the recovery of the identical vector *I*′. Once the vector is unmasked, the secret key *K* is generated.

### 3.8 Biometric extractor performance indicators

The False Rejection Rate (FRR) is calculated using Equation 10, which considers the probability of distortions in biometric features. False Rejection Rate (FRR) and False Acceptance Rate (FAR) are important metrics in biometric authentication. The FAR quantifies the possibility of an unauthorized person being erroneously granted access by the biometric system, whereas the False Rejection Rate (FRR) assesses the extent of inaccurate access denials for authorized users. To assess this probability, we shall examine two scenarios.

Let us assume that the biometric data scanning and processing produced a binary string $B^{*}=I.G_{x}+e^{*}$ , where the hamming weight of an error vector e* represents the potential differences between B* and a reference biometric set *B*. The number of non-zero positions in a vector e* is defined by the probability of a non-zero character occurring in e*. This probability represents the likelihood of a character in the codeword $B_{x}=I.G_{x}$ being distorted for both authorized and unauthorized users. However, these probabilities fluctuate between the two. Let us examine the initial scenario: Assume that the vector $B^{*}=I.G_{x}+e^{*}$ is owned by the authorized user. The probability of a single character distortion in *B_x* is denoted as P0. The formula can be used to estimate the value of FRR (Kuznetsov et al., 2022):


(10) $$FRR=1-\overset{t}{\underset{\dot{I}=0}{\sum }}C_{k}^{i}p_{0}^{i}{\left(1-p_{0}\right)}^{k-i}$$

The term ∑ represents the summation from *i* = 0 to *t*, where *t* is the maximum number of allowable errors or distortions in the biometric feature. $C_{k}^{i}$ denotes the combination or binomial coefficient, which calculates the number of ways to choose *i* distortions out of *k* total distortions. $p_{0}^{i}$ is the probability of having i distortions in the biometric feature. ${\left(1-p_{0}\right)}^{k-i}$ represents the probability of having k-i correct characters in the biometric feature. For each possible number of distortions (i) from 0 to t, the formula calculates the probability of that specific scenario occurring. The probabilities are then subtracted from 1 to get the overall False Rejection Rate.

Assume that the vector $B^{*}=I.G_{x}+e^{*}$ is possessed by an unauthorized user. The probability of a single character distortion is denoted as p1. The False Acceptance Rate (FAR) is determined using Equation 11, which evaluates the likelihood of unauthorized access. Subsequently, the value of FAR can be assessed in accordance with the prescribed formula (Kuznetsov et al., 2022):


(11) $$\mathrm{FAR}=\overset{t}{\underset{\dot{I}=0}{\sum }}C_{k}^{i}p_{1}^{i}{\left(1-p_{1}\right)}^{k-i}$$

The term ∑ represents the summation from *i* = 0 to *t*, where *t* is the maximum number of allowable errors or distortions in the biometric feature. $C_{k}^{i}$ denotes the combination or binomial coefficient, which calculates the number of ways to choose *i* distortions out of *k* total distortions. $p_{1}^{i}$ denotes the likelihood of occurrence *i* distortions in the biometric feature. ${\left(1-p_{1}\right)}^{k-i}$ denotes the likelihood of occurrence *k-i* correct characters in the biometric feature. For each possible number of distortions (i) from 0 to t, the formula calculates the probability of that specific scenario occurring. The probabilities are then summed up to get the overall False Acceptance Rate.

## 4 Experimentation

This section includes the hyperparameters we employed, the model configurations created, the outcomes of experiments conducted during the training, the evaluation of the construction of the Siamese Neural Network (SNN), the Binary string converter, the Cryptography Code, and, lastly, an explanation of the summary discussion.

### 4.1 Extracted features

**Figure 3:**
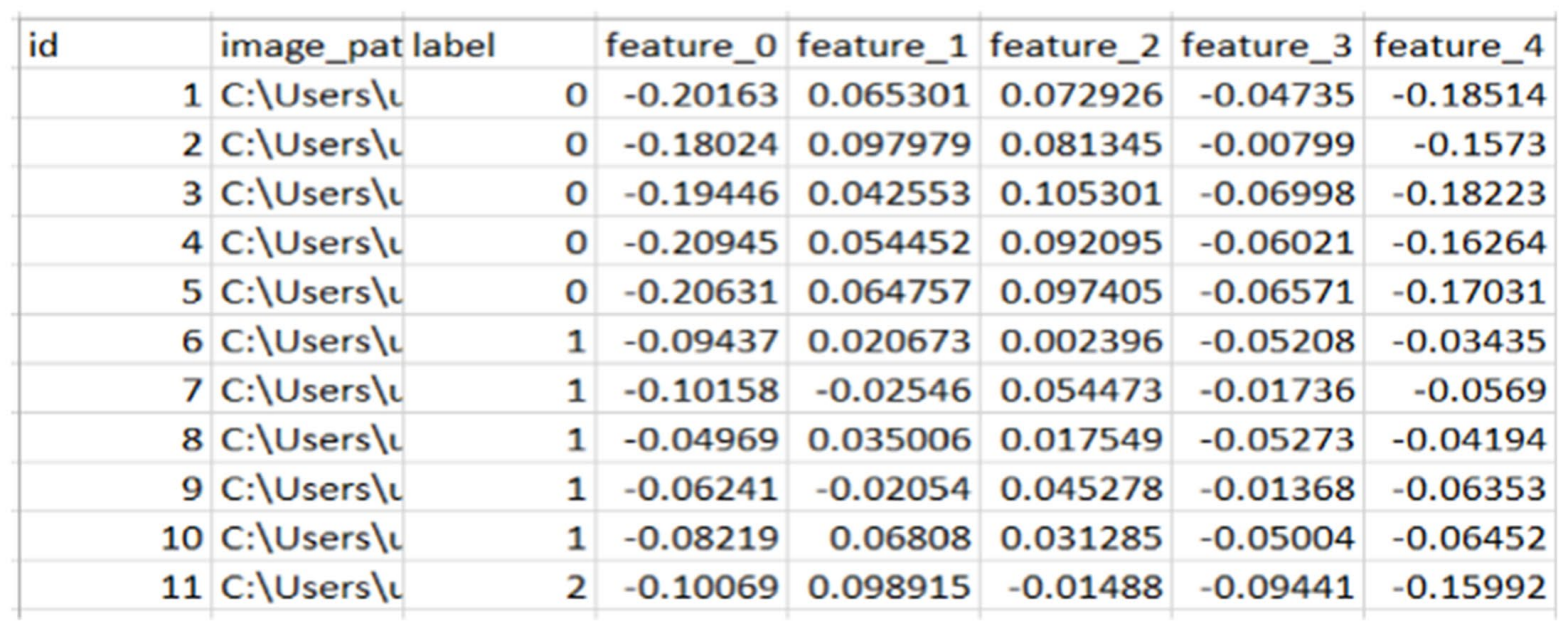
Sample of the extracted feature of face.

**Figure 4:**
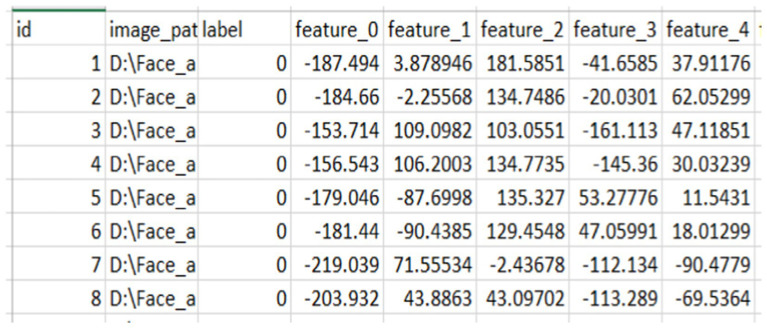
Sample of finger vein features.

From the above Figures 3, 4, we extracted 128 features from each image in column format and saved them as CSV for later use to fuse the face features with the finger vein. From here, we saved the images with their labels, image paths, and features.

### 4.2 The averaged and normalized finger vein features

**Figure 5:**
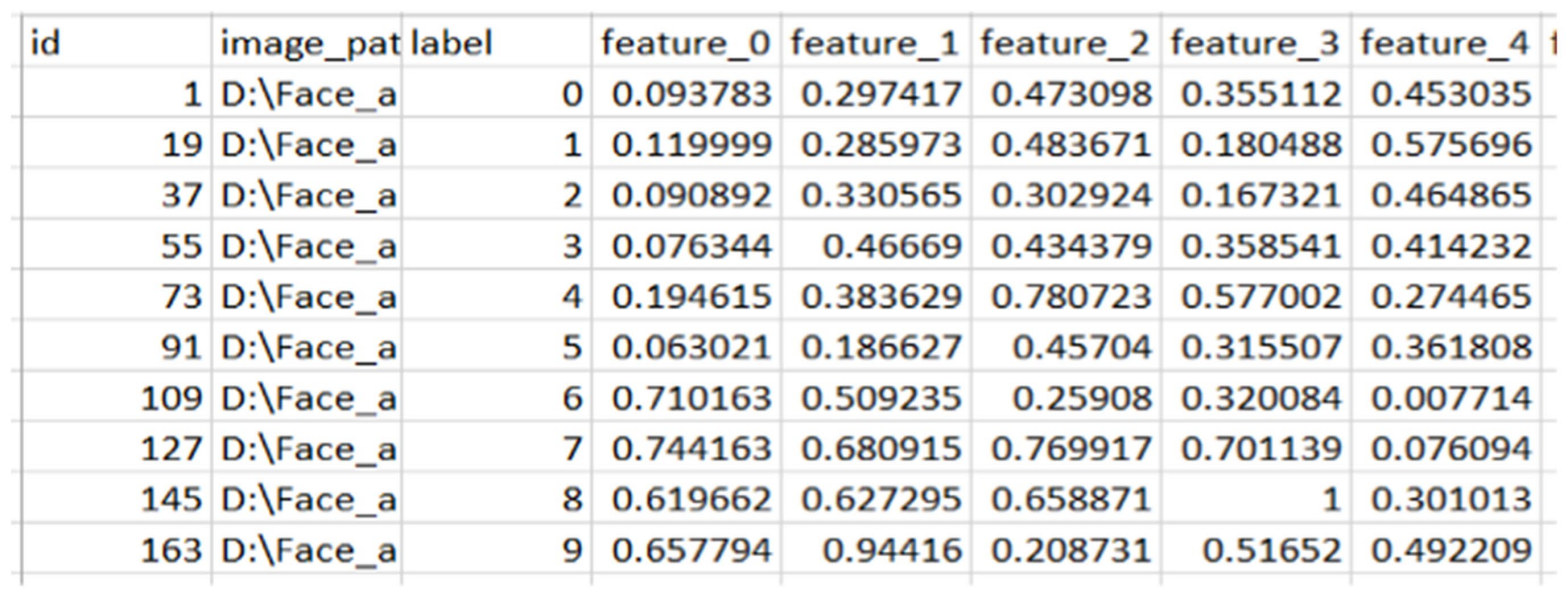
Sample of normalized and averaged finger vein features.

Before fusing face features, the averaged and normalized finger vein features for each class were calculated and saved as a separate CSV file, as shown in Figure 5. This process produces a representative feature vector that summarizes the attributes of samples within each class, reducing the dimensionality of the data and aiding in tasks such as visualization, grouping, and classification. Averaging features across multiple samples in a class minimizes noise and outliers, leading to more reliable and consistent class representations.

**Figure 6:**
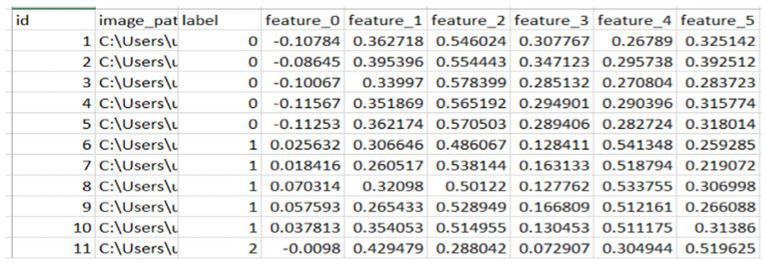
Sample feature of finger vein and face features of finger vein feature fused.

Specifically, for classes of faces and fingerprints defined as *n* × *n*, the first row of the finger vein class, which represents the first-class average of a finger vein, is combined with the first five rows of face features associated with that individual. This process continues incrementally, with each subsequent row of finger vein being fused with the next five rows of facial features until all data are processed. This method ensures that each averaged finger vein feature contributes to the corresponding face features, facilitating a comprehensive representation in the fused dataset, as illustrated in Figure 6. The resulting combined value reflects an incremental addition of features, enhancing the overall input before model training.

### 4.3 Dataset splitting summary

The distinct train-validation-test splitting method used is an 80-10-10 split. Table 1 summarizes the overall dataset splitting for the experiment and task.

**Table 1:** Dataset splitting.

Development	Training	Validation	Testing	Total
Exp	80%	10%	10%	1,500

The dataset split for each trial, with distinct categories and their associated values, is detailed in Table 1. It provides an overview of the data from the training, validation, and test sets used to build the model. So, in total, we used 425 datasets. We used 320 data to train the model, 40 for validation, and 45 for testing.

### 4.4 Hyperparameters used

We implemented a Siamese Neural Network with hyperparameter tuning using Keras and Kerastuner. The model includes adjustable parameters, batch size, regularization, dropout rate, and hidden layer size, using a custom distance loss function to train on triplet data. The SiameseHyperModel class manages hyperparameter optimization with RandomSearch and generates training batches. After training, the best model and its hyperparameters are evaluated and summarized. The model’s training process is configured based on 25 epochs, a batch size of 35, and a learning rate of 0.001. The ‘hard number = 10’ parameter helps the model handle challenging examples, while the Adam optimizer ensures efficient weight updates with adaptive learning rates. Validation steps monitor performance to prevent overfitting, with steps per epoch and steps per validation set to 15 and 2, respectively. These hyperparameters together influence the model’s learning behavior, convergence, and generalization performance.

## 5 Experimental result

This article contains the results and conclusions from a number of tests we ran to assess how well our deep learning-based SNN model performed for the fusion features. We also presented the findings from the SNN model with the code-based cryptography key generation. Initially, we provided the SNN outcomes, which played a crucial role in developing our cryptographic key generation model. The performance of the SNN model was evaluated by computing loss training and validation.

### 5.1 Result of SNN model

The component of the SNN model evaluation’s accuracy and loss is shown in this section. *Model Loss:* “Loss” measures the performance of a machine learning model by quantifying the difference between the predicted and actual outputs. It tracks the model’s learning progress during training, with values showing improvement across epochs.

#### 5.1.1 Accuracy

The accuracy of the model is measured using the similarity and dissimilarity of the features based on the label given, as shown in Table 2. If two images are similar, their difference is below the threshold value, and the images belong to the same person, but if the images are dissimilar, their difference is above the threshold, and they belong to different persons.

**Table 2:** Sigma similarity.

Threshold type	Sigma same	# of same pairs	Sigma diff	# of diff pairs	Sigma score
0.5 vector	tf.Tensor(0.92664933, shape = (), dtype = float32)	36	tf.Tensor(0.6359863, shape = (), dtype = float32)	32	0.613826
Expected value	tf.Tensor(0.9863281, shape = (), dtype = float32)	36	tf.Tensor(0.9250488, shape = (), dtype = float32)	32	0.388859

#### 5.1.2 Results of FAR and FRR for the code-based cryptography

Table 2 exhibits the empirical calculation of the *σ* ^ same, which is the average similarity of retrieved binary vectors for the same individual using multiple deep learning models. The provided data are utilized in the calculation of *p*_0_, which is *p*_0_ = 1− $\hat{\sigma \;}$ . Consequently, we determine that *p*_0_ is equal to 0.07. These numbers are used to compute *p*_0_ = 1− $\hat{\sigma }$ same (Kuznetsov et al., 2022). Similarly, we assess the value of *p*_1_’s based on the empirical results for $\hat{\sigma \;}$ diff. We have deduced that the value *p*_1_ = 0.37. These results provide significant novel insights into the performance of several deep learning models in generating binary vectors for biometric verification. They also indicate potential for additional progress and innovation in this field. The extraction approach relies on the use of code-based cryptosystems that employ a linear block code (*n*, *k*, *d*) = (2^m^, 2^m^ - mt, 2 *t* + 1) block (*n*, *k*, *d*) $=(2^{m},2^{m}-mt,2t+1$ ) with a fast-decoding process of polynomial complexity. The binary Goppa code with parameters (*n*, *k*, *d*) = $(2^{m},2^{m}-mt,2t+1$ ) for some m in Z^+^ is thought to be the best alternative (Kuznetsov et al., 2022). A comprehensive analysis and comparison of the False Acceptance Rate (FAR) and False Rejection Rate (FRR) were conducted to evaluate the system’s performance. The method being discussed relies on code-based cryptosystems that utilize a linear block (*n*, *k*, *d*) code that can be decoded rapidly with polynomial complexity. In our investigations, we generated binary strings of length *n* = 128 or *m* = 7. The parameters k and d of the Goppa codes for different *t* values are shown in Table 3. Table 3 also included the calculated values of FRR and FAR for estimations for different Goppa codes.

**Table 3:** FRR and FAR estimations for different Goppa codes.

*t*	*K*	*d*	SNN model	
			FAR	FRR
1	121	3	1.5754351486329734e-24	0.9990170515143107
5	79	15	4.100625196054598e-19	0.8905780902806273
10	65	19	2.6653196034529995e-14	0.28469187709689847
14	37	27	2.6218530171918587e-11	0.03455211265419574
17	9	35	2.0027487838478667e-09	0.003629338457096099

### 5.2 Comparative analysis of results

The outcomes of various other pertinent studies were compared with the study we conducted, as shown in Table 4. We provided a thorough comparison analysis of these findings below.

**Table 4:** A comparative analysis of the results.

Source	Methods and technologies	FRR	FAR	Additional Comments
Wang et al. (2021)	A secure biometric key generation mechanism via deep learning and its application	GAR = 98.47 and EER = 1.09 at fixed 1% FAR		During new enrollment, retrain the network to learn the mapping b\n new biometric image and binary code
Jana et al. (2022)	Neural fuzzy extractors for biometric authentication	2.5%–4.4%	2.5%–4.4%	Advances in iris-based biometric authentication have been made; however, the study of facial biometrics has not been expanded
Kuznetsov et al. (2022)	Code-based cryptographic extractor using Keras FaceNet face recognition	8.3%	7.4%	For a produced key length of 37 bits with a helper string. Furthermore, the code-based cryptosystems on which our extractor is built offer post-quantum level security
Our research	Cryptographic key generation via deep learning using Bi-modal biometric face and fingerprint	<3.4% for t between 14 and 17	<1%	For producing a key length of 128 bits using bimodal. Furthermore, the code-based cryptosystems on which our extractor is built offer post-quantum level security

In comparison to previous research, our work demonstrates significant improvements in biometric key generation by achieving a lower False Rejection Rate (FRR) of less than 3.4% and a False Acceptance Rate (FAR) of less than 1%, outperforming (Kuznetsov et al., 2022) FRR of 8.3% and FAR of 7.4%. Additionally, while it generated 37-bit keys, our research produced up to 51, providing enhanced security, especially with post-quantum cryptosystems. Moreover, by utilizing bimodal biometrics, our approach improves accuracy and robustness over single-modal systems, as expressed in Table 4, addressing the limitations of relying on a single biometric modality. This shows not only technical improvement but also compassion for users by ensuring higher security and usability.

## 6 Discussion of results

This section covers the model configuration, experimental findings, and hyperparameters utilized in the SNN model and cryptography code.

The proposed system utilizes deep learning models, including FaceNet for face feature extraction and VGG19 for fingerprint feature extraction, combined with a Siamese Neural Network (SNN) for feature fusion. While the computational complexity of these models is significant, our study primarily focused on security and accuracy metrics rather than execution time. Future work will include detailed performance analysis, measuring training and inference speed across different hardware platforms to ensure the system’s feasibility for real-time biometric authentication applications.

A dataset-splitting summary is shown in Table 1. Figure 6 lists the hyperparameters that were used: epoch, batch size, dropout, optimizer, activation function, length of the sequence, embedding dimension, loss, and train-test-split. One way to summarize the explanation of the experimental findings is as follows: Dataset Splitting: For the train and test SNN model, the dataset was divided into training, validation, and testing sets. Table 1 provides an overview of the data assigned to each set.

### 6.1 Model loss

**Figure 7:**
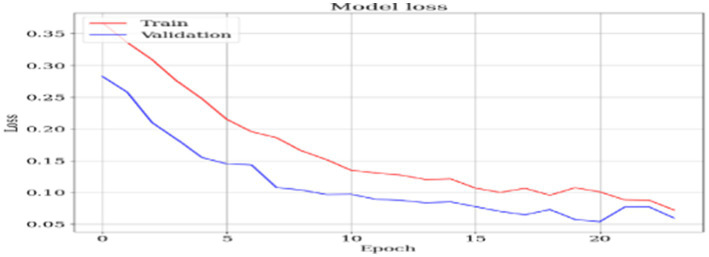
Model loss.

Figure 7 shows that as the epoch increases, the model performs better, as can be seen by the decreasing value of the loss measure. The image depicts a graph illustrating the “Model loss” progression throughout the training of a machine-learning model across many epochs. An epoch in machine learning refers to a single iteration over the entire training dataset. The graph illustrates two lines, with one line depicting the loss on the training set (in blue) and the other line indicating the loss on the validation set (in red). Each line in the graph demonstrates a decrease in loss as the number of epochs increases, suggesting that the model is effectively acquiring knowledge from the data. The x-axis is labeled “Epoch” and shows the progression of epochs from 1 to 25. The y-axis is labeled “Loss” and measures the magnitude of loss, with values from 0 to approximately 0.35.

### 6.2 Accuracy

This is calculated by computing a sigma score based on two values. The method is computing the average similarity in a Siamese network between photos of the same class (i.e., similar pairs) and images of different classes (i.e., dissimilar pairings). A formula cubes the difference between the average similarity of similar and different pairings (sigma_same and sigma_diff), multiplies it by sigma_same, and caps the result at zero to assess how well the network separated these pairs. The score measures the network’s ability to discriminate between similar and dissimilar pairings; higher values correspond to better performance. Class grouping of feature vectors (embeddings) and associated labels facilitates comparisons both within and across classes. The first step of the technique is to classify the data points into batches and then compute the average similarity (sigma_same) for comparable pairings within each batch. After converting the embeddings, a similarity function computes the similarity scores, which are then averaged and added together. To ensure an equal amount of comparisons from both classes, data points for dissimilar pairings are compared across batches. These different pairs’ similarity scores (sigma_diff) are computed and averaged. The average values of sigma_same and sigma_diff are then used to compute the sigma score, which gives an overall indication of how well the network can distinguish between similar and different embeddings. Overall, this accuracy estimator provides a way to evaluate the performance of a Siamese network by calculating average similarities for similar and dissimilar pairs based on a chosen converter function. It also computes a sigma score to quantify how well the network separates the two categories. From Table 2, based on the 0.5 threshold, the output is given. The average sigma same similarity and sigma difference similarity based on the above definition using a 0.5 threshold is 0.93 and 0.64, respectively.

Presently, FRR ≈ 25% and FAR ≈ 10% are considered acceptable markers of biometric identification based on a person’s facial picture. Meanwhile, facial recognition technology has advanced significantly in recent years. As per the National Institute of Standards and Technology (NIST) research (Libby and Libby, 2021), the optimal face recognition method exhibits an error rate of approximately 0.08% under ideal conditions. Furthermore, it is imperative to minimize the FAR values for biometric password generation systems as much as possible, ideally aligning them with the probability of password guessing. The situation in which the False Rejection Rate (FRR) is equal to or less than 10% is depicted in Table 3. The values shown are within the required range (<10%), with t varying from 10 to 17. The comparative analysis in Table 4 highlights the evolution of biometric key generation mechanisms, showing significant advancements in accuracy, security, and adaptability over time. Early methods had higher error rates, while more recent approaches, like our research, demonstrate much lower FRR and FAR rates, achieving less than 3.4 and 1%, respectively, for t between 10 and 17, with a strong up to 51-bit key length. Our biometric cryptographic key generation system ensures strong error tolerance and stability using Goppa codes, which correct variations in biometric data. Multimodal fusion significantly reduces spoofing risks by requiring multiple biometric traits for authentication. Unlike Rivest Shamir Adleman (RSA) and Elliptic Curve Cryptography (ECC), the McEliece cryptosystem provides post-quantum security, making it resistant to future quantum attacks. Compared to previous works, our method achieves a lower FRR (<3.4%) and FAR (<1%), significantly improving from previous FRR (8.3%) and FAR (7.4%). Additionally, our system generates 128-bit+ cryptographic keys, surpassing the 37-bit keys of earlier approaches, ensuring higher security and robustness.

**Figure 8:**
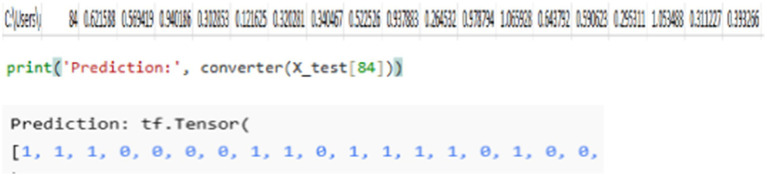
Binary string output.

Binary string output of the model: By feeding the model with a testing dataset from class 84, which includes the feature values shown in Figure 8, the model generates a binary string output. Using a binary string converter, the predicted values are transformed into a binary representation. The model’s prediction closely matches the expected output, demonstrating its accuracy in classification and conversion.

## 7 Conclusion and future work

This research demonstrates an advanced approach to cryptographic key generation by integrating deep learning with multimodal biometric data from face and finger vein modalities. Leveraging pretrained models such as FaceNet and VGG19, along with a Siamese Neural Network (SNN) and fuzzy extractors, the system achieves high accuracy and robustness, with a False Acceptance Rate (FAR) below 1% and a False Rejection Rate (FRR) below 3.4%. Incorporating Goppa code-based cryptographic systems ensures post-quantum security, making the approach highly resilient. Future work will focus on optimizing neural network architectures, integrating additional biometric modalities, and exploring quantum-resistant algorithms to address emerging challenges. Real-time implementation, enhanced scalability, and adaptability to diverse user environments will also be prioritized to improve usability and robustness. Future work will focus on testing with larger and more diverse biometric datasets to further validate the robustness of the approach. Additionally, efforts will be directed toward optimizing neural network architectures, integrating additional biometric modalities, and exploring quantum-resistant algorithms. Real-time implementation, enhanced scalability, and adaptability to diverse user environments will also be prioritized to improve usability and robustness. While the computational complexity of these models is significant, our study primarily focused on security and accuracy metrics rather than execution time. Future work will include detailed performance analysis, measuring training and inference speed across different hardware platforms to ensure the system’s feasibility for real-time biometric authentication applications.

## Data Availability

The original contributions presented in the study are included in the article/Supplementary material, further inquiries can be directed to the corresponding author.
