# Supplementary figures and images for: Cryptographic key generation using deep learning with biometric face and finger vein data
Source: Front Artif Intell. 2025 Apr 29;8:1545946. doi: 10.3389/frai.2025.1545946 (PMC12069345; doi:10.3389/frai.2025.1545946)

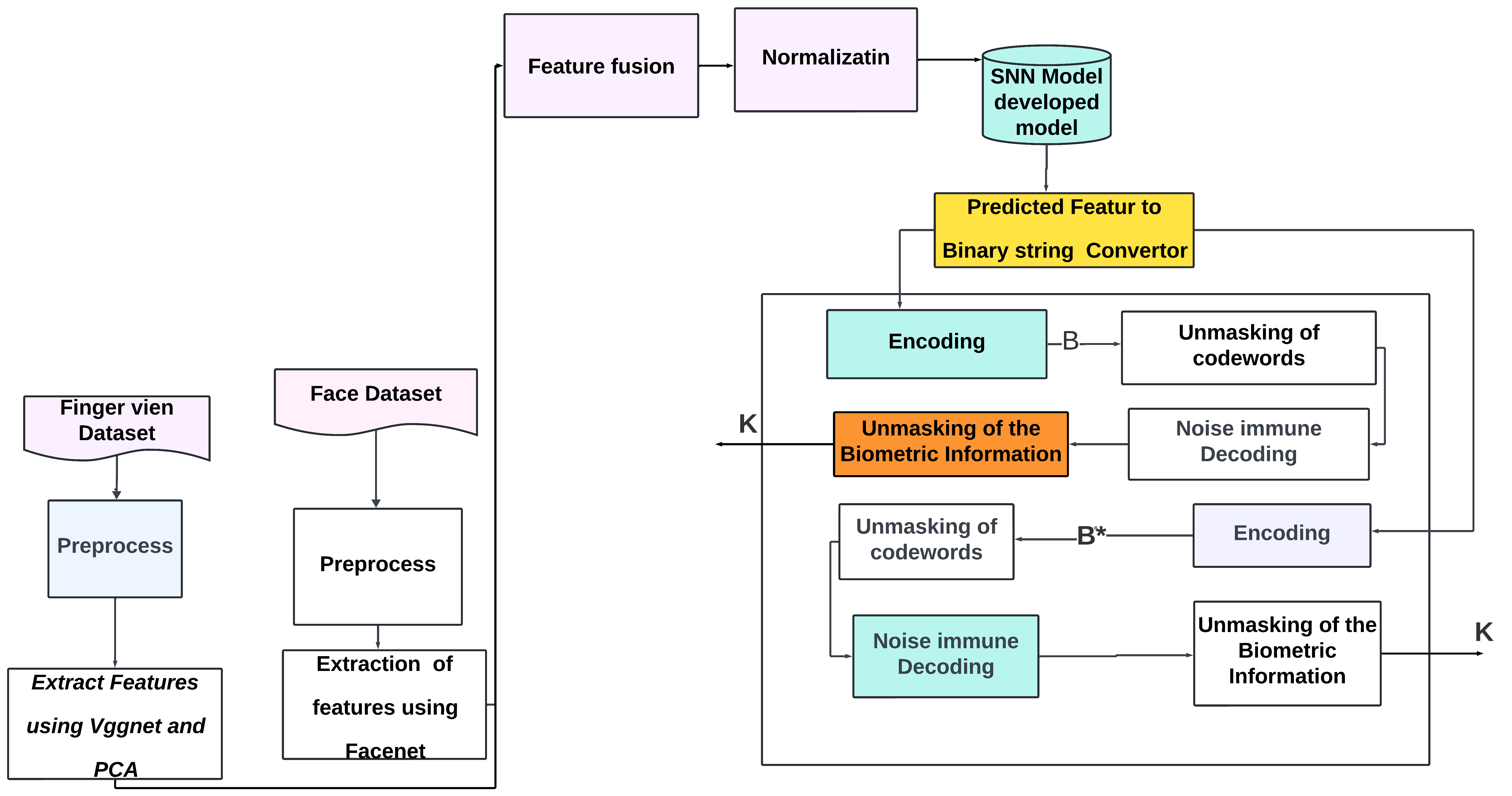

Supplement: Supplementary file 5 [file Image_1.png]

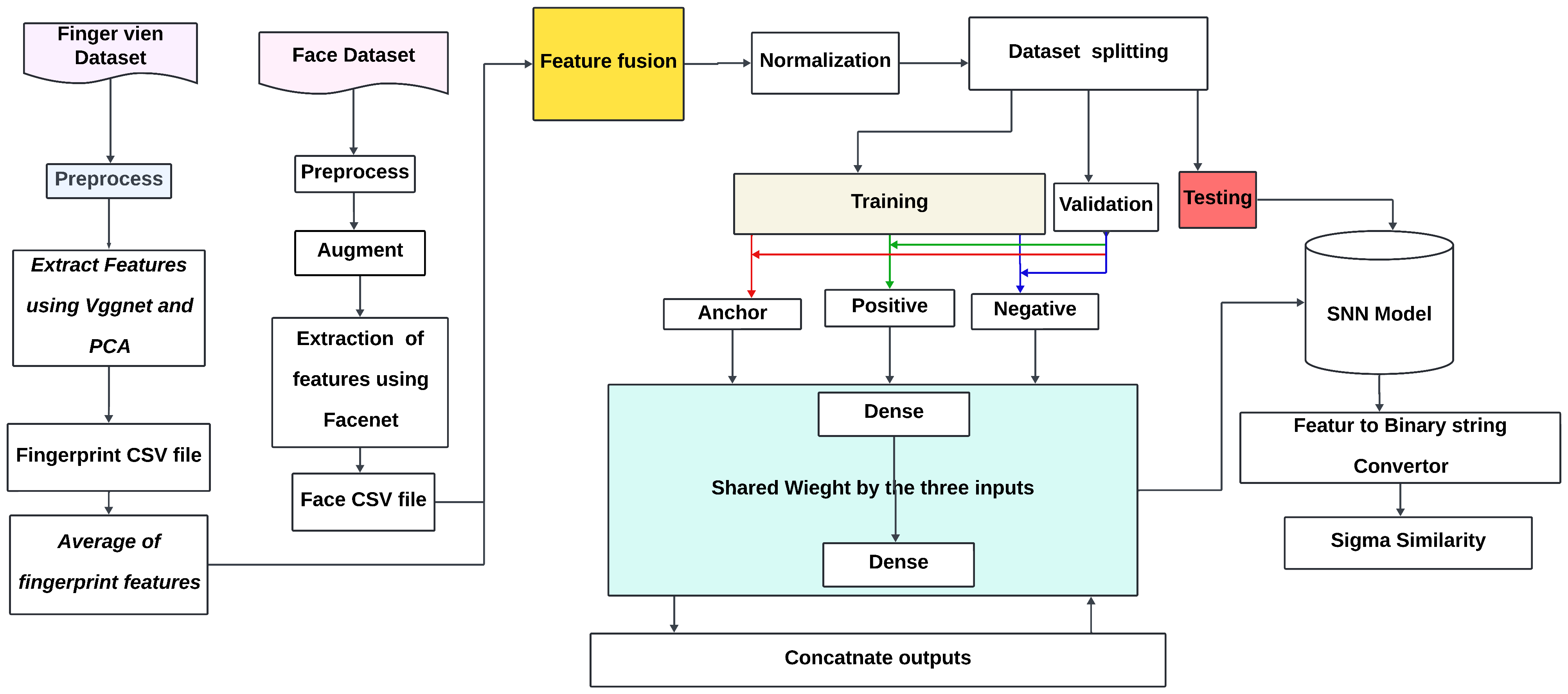

Supplement: Supplementary file 6 [file Image_2.png]
